# Gait Training and Ankle Dorsiflexors in Cerebral Palsy

**DOI:** 10.15844/pedneurbriefs-29-3-5

**Published:** 2015-03

**Authors:** J. Gordon Millichap

**Affiliations:** 1 Division of Neurology, Ann & Robert H. Lurie Children's Hospital of Chicago, Chicago, IL; 2 Departments of Pediatrics and Neurology, Northwestern University Feinberg School of Medicine, Chicago, IL

**Keywords:** Cerebral Palsy, Coherence, Development, Gait

## Abstract

Investigators at University of Copenhagen, Denmark, evaluated whether 4 weeks of 30 min daily treadmill training with an incline may facilitate corticospinal transmission and improve control of the ankle joint in 16 children, aged 5-14 years, with cerebral palsy.

Investigators at University of Copenhagen, Denmark, evaluated whether 4 weeks of 30 min daily treadmill training with an incline may facilitate corticospinal transmission and improve control of the ankle joint in 16 children, aged 5-14 years, with cerebral palsy. Gait training was accompanied by significant increases in gait speed, incline on the treadmill, the maximal voluntary dorsiflexion torque, and the weight exerted on the heel. EMG-EMG coherence in beta and gamma frequency bands recorded from the tibialis anterior increased significantly. Daily intensive gait training increases beta and gamma oscillatory drive in ankle dorsiflexor motor neurons and improves toe lift and heel strike in children with cerebral palsy and corticospinal dysfunction, especially at <10 years of age. [1]

COMMENTARY. Cerebral palsy with toe-walking is hemi- or diplegic [1]. Rarely, an asymmetric toe-walking can be dystonic and transient [2] and an explanation for “idiopathic” toe walking. Under 2 years of age, toe walking may not be pathologic; when persistent after the age of 2 years and in the absence of neurological or orthopedic abnormalities, toe- walking is referred to as idiopathic. The type of treatment is based on age and severity of the abnormality. An equinus contracture can develop, sometimes leading to casting, and/or operative treatment. In studies comparing casting and operative treatment of children with idiopathic toe walking, no significant differences between groups were found [3]. Treadmill interventions in children up to 6 years of age with Down syndrome, at risk of motor delay, led to earlier onset of independent walking [4]. Treadmill intervention may have a general effect on motor development in both children with corticospinal tract dysfunction and in those at risk of motor delay.
